# Evaluation of different milking practices for optimum production performance in Sahiwal cows

**DOI:** 10.1186/2055-0391-56-13

**Published:** 2014-08-07

**Authors:** Naveed Aslam, Muhammad Abdullah, Muhammad Fiaz, Jalees Ahmad Bhatti, Zeeshan Muhammad Iqbal, Nasrullah Bangulzai, Chang Weon Choi, Ik Hwan Jo

**Affiliations:** Department of Livestock Production, Faculty of Animal Production and Technology, University of Veterinary and Animal Sciences, Lahore, Pakistan; Department of Livestock Production and Management, Pir Mehr Ali Shah Arid Agriculture University Murree Road Shamsabad, Rawalpindi, Pakistan; Department of Livestock Management, Lasbela University of Agriculture, Water and Marine Sciences Balochistan, Karachi, Pakistan; Department of Animal Resources, International Research Center for Eradication of Poverty and Hunger, Daegu University, Gyeongsan, South Korea

**Keywords:** Sahiwal cows, Machine milking, Milking frequency, Milk yield, Composition

## Abstract

The production performance of multiparous lactating Sahiwal cows (n = 24) was evaluated according to both milking frequency and method. Selected animals were randomly divided into four groups containing six animals each under a completely randomized design. Cows in groups A & B were milked by the hand milking method three times per day, respectively. Similarly, cows in groups C & D were milked by the machine milking method two and three times per day, respectively. All animals were maintained under uniform feeding and management conditions. Dry matter intake was high in animal groups milked three times per day, and it remained unchanged between the hand and machine milking methods. Milk yield was higher (P < 0.05) in cows milked three times compared to those milked twice per day, and it did not differ between hand and machine milking methods. Milk fat percentage was higher (P < 0.05) in cows milked twice per day compared to those milked three times using both machine and hand milking methods. The percentage of total solids showed a similar pattern as the fat percentage. However, percentages of protein, lactose, and non-fat solids in milk were not significantly different (P > 0.05) among the treatment groups. Collectively, the results show that milking three times per day instead of twice at 8-hour intervals can enhance milk yield in Sahiwal cows using both hand and machine milking methods.

## Background

Demand for milk in Pakistan is rising due to an increasing human population and higher inclination towards consumption of milk and milk products. Milk is the largest commodity amongst dairy products, making Pakistan the 4^th^ largest milk-producing country in worldwide. A total of 49.5 billion kg of milk was produced during the 2012-13 year [1]. Major sources of milk in Pakistan are cattle and buffaloes, as the country is blessed with suitable tropical breeds of dairy cattle. Sahiwal is one such renowned tropical dairy cattle breed due to its excellent heat and tick resistance [2, 3]. As such, the performance of this breed in a tropical environment is also better than other cattle breeds due its better resistance to tick-borne diseases [4].

Cow milking is considered to be one of the most laborious and time-consuming activities at livestock farms. Additionally, considerable cost goes into this major farm operation. As a result, machine milking was invented to improve labor efficiency due to growing costs [5]. Machine milking has been shown to have the potential to increase milk production by up to 12%, reduce labor by up to 18%, and improve dairy cow welfare.

Increasing milking frequency from twice to three times per day may correspondingly increase milk yield from 6 to 25% during complete lactation as well as improve udder health by reducing the somatic cell count (SCC) [6–8]. The chance of clinical mastitis has also been reported to be lower in cows milked three times or more than in those milked only twice per day [9]. Milk yield can be increased by machine milking, which has beneficial effects on udder health [10, 11]. Moreover, milking three times per day has been shown to increase milk yield by up to 14% compared to milking only twice per day [12]. The literature is limited regarding the effects of milking frequency as well as machine milking on milk yield, composition, and udder health of Pakistani cattle. Therefore, this study investigated the optimal milking frequency and milking method for improved production performance in Sahiwal cows.

## Methods

### Precinct of study

The study was conducted at the Livestock Experiment Station Jahangirabad, Khanewal. This station is situated in south central irrigated Punjab, Pakistan. Its geographical coordinates are 30° 18' 0" North, 71° 56' 0" East. Jahangirabad town in Khanewal District is well known as the homeland of Sahiwal cows, a renowned tropical dairy cattle breed in Pakistan [2, 3, 13]).

### Experimental animals and treatment groups

Twenty-four lactating Sahiwal cows in their 3^rd^ to 4^th^ parity after parturition with similar average bodyweight, conditions, and production performance were separated into groups A, B, C, & D. Cow milking was carried out by the hand and machine milking methods twice or three times a day, as shown in Table 1. The animals were kept for a period of 90 days, excluding a 15-day adjustment period. The cows were also properly tagged and treated for internal and external parasites. All animals were kept in separate pens under identical conditions.

**Table 1:** **Layout of experiment**

Treatment groups	Replicates	Milking method	Frequency of daily milking
**A**	6	Manual	Twice
**B**	6	Manual	Thrice
**C**	6	Machine	Twice
**D**	6	Machine	Thrice

### Feeding of animals

Animals were given standard diet: daily green fodder (Maize) @ 40 kg per cow, wheat straw @ 4 kg per cow, and Anmol wanda (concentrate consisting of CP 16 and 68% TDN) @ 1.0 kg per 2.5 liters of milk produced by each cow. Composition of Anmol Wanda is given in Table 2. Clean premises were made available to the experimental cows, and animals had *ad limitum* access to fresh clean water.

**Table 2:** **Ingredient and nutrient composition**

S. No.	Ingredient (s)	Inclusion level (%)
1	Cotton seed Cake	18
2	Maize gluten 30	15
3	Canola meal	10
4	Maize grain broken	14
5	Wheat Bran	25
6	Molasses	16
7	Minerals mixture*	2
Total		100
CP (%)		16
TDN (%)		68

*100 Kg minerals mixture included DCP 70.81 kg, NaCl 18.91 kg, MgSO_4_ 8.64 kg, FeSO_4_ 0.89 kg, MnSO_4_ 0.49 kg, ZnSO_4_ 0.22 kg, CuSO_4_ 0.03 kg, KI 8.77 g, CoCl_2_ 0.89 g, and NaSiO_3_ 1.50 g.

### Milking of cows

Cows in groups A & B were subjected to the hand milking method, whereas cows in groups C & D were milked using a portable milking machine. However, groups A & C were milked twice per day at 12-hour intervals at 3:00 and 15:00 hours, whereas groups B & D were milked three times per day at 8-hour intervals at 2:00, 10:00, and 18:00 hours. Prior to milking, the udder of each cow was thoroughly washed with moderate warm water & dried properly. The milking machine was thoroughly washed with acid and alkali solutions in lukewarm water.

### Data recording and parameters

Feed intake of each experimental animal was recorded daily. Milk yields of all cows were recorded daily using a calibrated spring balance. To screen for mastitis, a few milk were tested prior to milking. Milk samples were collected from all cows every 2 weeks for milk composition analysis. The following parameters were studied to determine the response to different treatments.

1. Dry matter intake (kg)
2. Milk yield (kg)
3. Milk protein%
4. Milk fat%
5. Lactose%
6. Total solids%

### Laboratory analysis

Analyses were performed in the Food & Nutrition and Quality Operation Labs at the University of Veterinary and Animal Sciences (UVAS), Lahore. Dry matter content of feed samples was determined according to the procedures of [14]). Milk composition analysis was carried out using a Lactoscan-S Milk Analyzer (50 W, Milkotronic Ltd., Bulgaria) in WTO-Quality Operation Labs at the UVAS, Lahore for the following milk constituents: milk fat, non-fat solids, milk protein, lactose, and total solids.

### Statistical analysis

Data were analyzed using ANOVA techniques [15] under a Completely Randomized Design (CRD) using SAS 9.1.3 portable software. Differences among treatment means were tested by DMRt. The mathematical model was:


where

**Y**ij = each observation on **i**^th^ treatment due to **j**^th^ animal.

**μ** = overall mean.

**τ**i = effect of **i**^th^ treatment (Σ τi = 0 and i = 1, 2, 3, 4).

**ϵ**ij = random error associated with **i**^th^ treatment and **j**^th^ animal with the restriction that variance **σ**_**2**_ and mean zero.

## Results and discussion

### Dry Matter Intake

Dry matter intake (DMI) in cows is shown in Table 3. Cows milked three times daily showed higher DMI compared to those milked twice per day. However, DMI was not significantly different between hand and machine milking methods. The higher DMI consumption of cows milked three times per day can be attributed to higher milk yield, which increases nutrient demands. In support of the current results, [16] previously studied the effects of milking frequency on production efficiency in Holstein cows and found that milking three times daily increased milk yield and DMI. In another study, [17] asserted that DMI increases (p < 0.05) with greater milking frequency. The current results are also supported by previous works ([16, 18, 19]) in which increased DMI was shown to be accompanied by higher milk yield due to greater milking frequency. Milk yield is an indicator of feeding behavior as increased milk yield is positively correlated with DMI [20].

**Table 3:** **Effect of milking frequency & method on dry matter intake and milk yield in Sahiwal cows**

Parameters	Hand milking twice (A)	Hand milking thrice (B)	Machine milking twice (C)	Machine milking thrice (D)
Dry matter intake (Kg/day)	7.50 ± 0.18^b^	9.30 ± 0.11^a^	7.90 ± 0.15^b^	9.50 ± 0.15^a^
Milk yield (Kg)	9.08 ± 0.15^b^	11.50 ± 0.18^a^	9.25 ± 0.21^b^	11.75 ± 0.28^a^

Different superscripts in same row differ significantly (P < 0.05).

### Milk yield

Average daily milk yields are presented in Table 3. Milk yield was higher (P < 0.05) in cows milked three times daily compared to cows milked twice. Milk production between the machine and hand milking treatment groups was not significantly different (P > 0.05). However, higher milking frequency significantly (P < 0.05) enhanced milk yield.

Our observation of an association between enhanced milking frequency and greater milk yield has been substantiated by the literature. In a previous study, [7] reported that milk yield increased by up to 10.4% in cows milked three times per day compared to those milked twice per day. Similarly, [21] showed that milking three times daily increased milk yield in cows by up to 18%. In another study, [22] showed that the yield of energy corrected milk was higher in cows milked three times per day compared to those milked twice per day.

Similarly, [23] affirmed that milking three times per day rather than twice increased milk yield in crossbred (different blood ratios among Friesian, Hariana, Brown Swiss, and Jersey breeds) cows, but at the cost of their body condition. The higher milk yield in cows milked three times per day might be due to the fact that milk secretion is a continuous process resulting in gradual elevation of internal udder pressure. Thus, more frequent milking might reduce internal udder pressure and consequently stimulate milk-secreting cells to operate at full capacity for a longer time.

### Milk composition

The impacts of milking frequency and method on milk composition in Sahiwal cows are shown in Table 4. Milk fat percentage was higher (P < 0.05) in cows milked twice compared to those milked three times per day. However, milking method did not influence fat percentage in milk. Similarly, the percentage of total solids was higher in milk from cows milked twice per day compared to those milked three times.

**Table 4:** **Effects of milking frequency & method on milk composition in Sahiwal cows**

Parameters	Hand milking twice (A)	Hand milking thrice (B)	Machine milking twice (C)	Machine milking thrice (D)
Protein%	3.35 ± 0.06	3.25 ± 0.06	3.35 ± 0.06	3.22 ± 0.08
Fat%	3.68 ± 0.04^a^	3.45 ± 0.03^b^	3.70 ± 0.04^a^	3.50 ± 0.04^b^
Lactose%	4.82 ± 0.06	4.87 ± 0.02	4.90 ± 0.04	4.92 ± 0.02
Total Solids%	12.7 ± 0.04^a^	12.4 ± 0.04^b^	12.7 ± 0.04^a^	12.4 ± 0.04^b^

Different superscripts in same row differ significantly (P < 0.05).

However, the percentages of protein and lactose in milk were not significantly different (P > 0.05) among cows in the different treatment groups. Milk fat content has been shown to be affected by milking frequency [7, 24, 25]). Similarly, cows milked three times per day produce milk with a lower fat content compared to those milked once a day during early lactation [26]. The negative effect of frequent milking on fat content can be attributed to increased air exposure due to frequent milking, enzymatic activity of fatty acid syntethase, and increased production of short-chain fatty acids [7]. Another factor might be the shortened time for fat synthesis in the case of 8-hour intervals. The high percentage of total solids in milk from cows milked twice daily might be due to a high fat percentage.

## Conclusion

It can be concluded that milking three times instead of only twice per day at 8-hour intervals can enhance milk yield in Sahiwal cows using both hand and machine milking methods.
